# 297 Pressure in the Operating Room (OR): another Possible Contributor to Hospital Acquired Pressure Injuries (HAPIs)

**DOI:** 10.1093/jbcr/irad045.272

**Published:** 2023-08-29

**Authors:** Thomas Milazzo, Robert Cartotto, Hannah Loo, Alan D Rogers

**Affiliations:** Ross Tilley Burn Centre, University of Toronto, Toronto, Ontario; Ross Tilley Burn Centre, University of Toronto, Toronto, Ontario; Ross Tilley Burn Centre, University of Toronto, Toronto, Ontario; Ross Tilley Burn Centre, University of Toronto, Toronto, Ontario

## Abstract

**Introduction:**

Burn patients are at risk for HAPIs. An unexamined factor that may contribute to HAPI development is the effect of pressure from the OR table. Burn patients often require multiple prolonged surgical procedures. The purpose of this study was to measure pressure on the buttocks and sacral area during burn OR procedures under general anesthesia (GA). This has not been previously assessed in the burn literature.

**Methods:**

Prospective study of consecutive adult burn patients admitted to an ABA-verified burn center who required surgery under GA between 06/01/22 and 08/12/22. We studied only cases that were supine, including those with both legs down (BLD), one leg suspended (one leg up: 1LU) or both legs suspended (two legs up: 2LU). Pressures on the buttocks and sacral area were measured using a commercial sensor mat which sent continuous real-time pressure measurements wirelessly to a laptop computer. Thousands of individual pressure measurements were integrated and organized to show average and peak pressures over repetitive 10-minute intervals during the entire operative procedure. Also, a continuous visual color-coded map of the sacral and buttock areas showing high ( >75 mmHg-red), medium (10-75 mmHg -yellow) and low ( < 10 mmHg -blue/green) pressures was created for each procedure. Results are shown as mean ± SD.

**Results:**

Recordings were completed in 41 procedures (35 acute excision & grafting, 5 reconstructive, 1 tracheostomy) among 28 patients ( 48 ± 17 yrs, % TBSA burn 19 ± 17, weight 80 ± 20 kg, BMI 27 ± 6). During BLD, over 125 ± 81 min, the average pressure (P_ave_) was 47 ± 7 mmHg and peak pressure (P_peak_) reached 82 ± 23 mmHg. During isolated 1LU periods, P_ave_ was 53 ± 9 mmHg over 74 ± 58 minutes with a P_peak_ of 93 ± 23 mmHg and during isolated 2LU, P_ave_ and P_peak_ were respectively 55 ± 10 mmHg over 78 ± 40 min and 98 ± 23 mmHg. Both P_ave_ and P_peak_ increased significantly from procedures with BLD to 1LU to 2LU (p< 0.001). P_ave_ crept steadily upwards during both BLD and 1LU or 2LU procedures (Figure). We observed that P_ave_ had a positive relationship with weight, regardless of operative position.

**Conclusions:**

Prolonged moderate to high pressures are exerted on the sacral and buttock areas, especially with one or both legs suspended, during burn surgery. These novel observations suggest that pressure from the OR table could contribute to HAPI development.

**Applicability of Research to Practice:**

With this risk identified, interventions to lower or interrupt pressure exposure during burn surgery should be identified next.

